# Susceptibility of the Oral Commensal Bacterium *Streptococcus sanguinis* to ZnO Nanoparticles

**DOI:** 10.3390/ijms27062782

**Published:** 2026-03-19

**Authors:** Raphaelle Emram, Ronit Vogt Sionov, Adi Aharoni, Sarah Gingichashvili, Noa E. Cohen, Vitaly Gutkin, Moshe Amitay, Asaf Wilensky, Doron Steinberg, Rawi Assad

**Affiliations:** 1 Faculty of Dental Medicine, Institute of Biomedical and Oral Research (IBOR), The Hebrew University of Jerusalem, Ein Kerem Campus, Jerusalem 9112102, Israel; raphaell.emram@mail.huji.ac.il (R.E.); ronit.sionov@mail.huji.ac.il (R.V.S.); adi.ahaorni@mail.huji.ac.il (A.A.); sophiko.gingichashvili@mail.huji.ac.il (S.G.); dorons@ekmd.huji.ac.il (D.S.); 2 Department of Periodontology, Hadassah Medical Center, Faculty of Dental Medicine, Hebrew University of Jerusalem, Jerusalem 91120, Israel; 3 School of Software and Electrical Engineering, Azrieli College of Engineering, Jerusalem 9103501, Israel; noace@jce.ac.il; 4 Department of Bioinformatics, Jerusalem College of Technology, Jerusalem 9548370, Israel; mosh9900@gmail.com; 5 Unit for Nano Characterization, The Harvey M. Krueger Family Center for Nanoscience and Nanotechnology, The Hebrew University of Jerusalem, Edmond J. Safra Campus, Jerusalem 9190401, Israel; vitalyg@savion.huji.ac.il

**Keywords:** antibacterial, antibiofilm, ROS production, *Streptococcus sanguinis*, ZnO nanoparticles

## Abstract

*Streptococcus sanguinis* (*S. sanguinis*) is an oral commensal and early colonizer of the tooth surface that contributes to dental biofilm homeostasis. Zinc oxide nanoparticles (ZnO NPs) are often incorporated into dental restorative materials to enhance mechanical performance and confer antibacterial properties; however, their effects on *S. sanguinis* have not been thoroughly studied. Here, we investigated the antimicrobial and antibiofilm efficacy of ZnO NPs against this bacterial species. ZnO NPs exhibited a minimal inhibitory concentration (MIC) of 100 µg/mL and caused rapid, dose-dependent suppression of intracellular ATP levels and overall metabolic activity within 2–4 h of exposure. ZnO NPs induced reactive oxygen species (ROS) production in a dose-dependent manner. The free radical scavenger α-tocopherol partly prevented the antibacterial effect of ZnO NPs, suggesting that lipid peroxidation contributes to ZnO NP-mediated toxicity, although it is not the sole mechanism involved. Short-term exposure (2 h) to ZnO NPs did not significantly affect membrane integrity or cellular morphology, whereas prolonged treatment (24 h) resulted in pronounced membrane permeabilization, membrane hyperpolarization, and cellular swelling. Computational morphometric analyses of high-resolution scanning electron microscopy (HR-SEM) images of planktonic growing bacteria after a 24 h treatment confirmed a significant, dose-dependent increase in cell surface area and surface roughness. Importantly, ZnO NPs also reduced the metabolic activity and compromised the structural integrity of mature, preformed biofilms. Collectively, these findings demonstrate that ZnO NPs exert antimicrobial and antibiofilm effects against *S. sanguinis* through early metabolic inhibition associated with oxidative stress followed by progressive membrane dysfunction.

## 1. Introduction

The development and progression of oral infectious diseases, such as dental caries and periodontitis, are fundamentally driven by the formation of complex microbial biofilms [1,2]. Within this ecosystem, *Streptococcus sanguinis* (*S. sanguinis*) is a key Gram-positive commensal and an early colonizer of the tooth surface. As a member of the mitis group, it provides the essential structural biofilm foundation for the subsequent attachment of other microorganisms, thereby guiding microbial succession [3,4]. Commonly associated with healthy dental plaque, *S. sanguinis* contributes to oral homeostasis by antagonizing cariogenic pathogens, such as *Streptococcus mutans* (*S. mutans*), primarily through the production of hydrogen peroxide (H_2_O_2_) [5,6,7]. However, its clinical significance is dual: despite its beneficial role in the oral cavity, it is a leading cause of infective endocarditis when it gains access to the bloodstream and colonizes the cardiac endothelium [6,8,9].

To manage oral dysbiosis and the overgrowth of pathogenic microorganisms, nanoparticles (NPs) have emerged as alternatives to traditional drug dosage forms, such as antibiotics, which often face limitations due to the development of bacterial resistance [10]. Among these, ZnO NPs are particularly notable for their broad-spectrum antimicrobial activity and chemical stability [11,12,13,14]. The antibacterial effect of ZnO NPs is multifaceted and includes the generation of reactive oxygen species (ROS), physical disruption of bacterial membranes, and the release of toxic Zn^2+^ ions that oxidize the amino acid chain, resulting in protein denaturation, impairment of metabolic pathways, and inhibition of bacterial growth [12,15,16,17,18,19,20].

While our previous research elucidated the mechanisms by which ZnO NPs inhibit the viability, biofilm formation, and extracellular polysaccharide (EPS) production of the cariogenic bacterium *S. mutans* [21], the specific physiological and metabolic responses of *S. sanguinis* to these nanomaterials remain to be elucidated. Previous studies have shown that *S. sanguinis* is sensitive to ZnO NPs prepared by green synthesis using the Mexican plant *Dysphania ambrosioides* [22].

This study aimed to provide mechanistic insight into the antibacterial and antibiofilm activities of ZnO NPs against *S. sanguinis*. Specifically, we investigated the modes of action against this early colonizer by assessing changes in its metabolic activity, membrane integrity, and structural stability in both planktonic and biofilm states. To objectively quantify ZnO NP-induced morphological changes at the single cell level, high-resolution scanning electron microscopy (HR-SEM) images were analyzed using a computational pipeline integrating deep learning-based segmentation (Cellpose), geometric feature extraction, and surface texture quantification. This approach enabled systematic assessment of bacterial morphology, size distribution, and membrane disruption. By establishing the structural and metabolic benchmarks of ZnO NPs-induced effects, this research supports the rational development of ZnO NPs as effective bioactive agents for preventive and restorative dental applications.

## 2. Results

### 2.1. ZnO NPs Inhibit Bacterial Growth and Metabolic Activity

**Figure 1:**
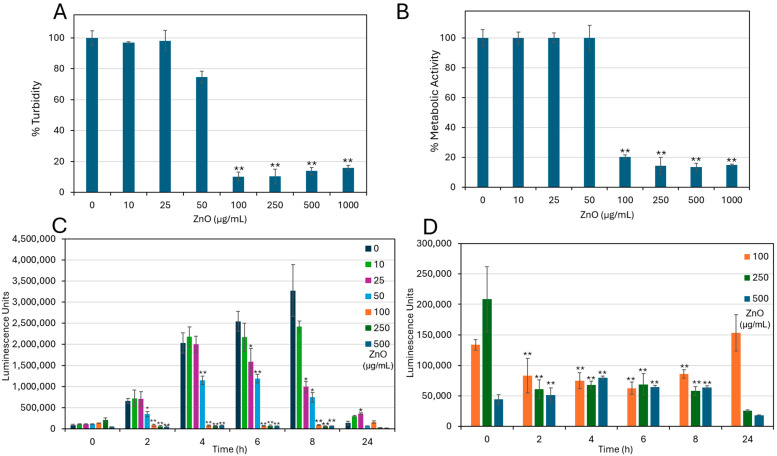
ZnO NPs inhibit the planktonic growth of *S. sanguinis*. (**A**) Planktonic growth of *S. sanguinis* following a 48 h incubation with indicated ZnO NPs concentrations as determined by turbidity. (**B**) Metabolic activity of planktonic growing bacteria after a 48 h incubation with ZnO NPs as determined by MTT metabolic assay. The control bacteria incubated for 48 h in the absence of ZnO NPs were set to 100%. Data are expressed as mean ± SD (*n* = 3). ** *p* < 0.001 against their respective control. (**C**) Intracellular ATP levels of *S. sanguinis* were monitored over a period of 24 h in the absence or presence of increasing concentrations of ZnO NPs, as determined by the BacTiter Glo viability assay. (**D**) Intracellular ATP levels in ZnO NP-treated samples, shown in (**C**), are presented to visualize temporal changes at high ZnO NP concentrations. Data are presented as mean ± SD of triplicates (*n* = 3). At each time point, the treated group was compared to its corresponding time-matched control using Bonferroni-corrected *t*-tests. * *p* < 0.0083, ** *p* < 0.001 compared to control.

The antimicrobial effect of ZnO NPs against *S. sanguinis* was initially studied to determine the minimum inhibitory concentration (MIC). A significant reduction (F(7, 16) = 612.23, *p* < 0.001) in bacterial growth (88.6 ± 0.6% inhibition) was observed at ZnO NPs concentrations of 100 μg/mL and above, establishing 100 μg/mL as the MIC (Figure 1A). In agreement with these findings, the metabolic activity of planktonic-growing bacteria was significantly impaired (F(7, 16) = 612.23, *p* < 0.001) at 100 µg/mL, showing a 79.8 ± 0.3% decrease compared with untreated controls (Figure 1B). No significant inhibition of bacterial growth was detected at 50 µg/mL, indicating a specific sensitivity threshold of *S. sanguinis* to ZnO NPs at 100 μg/mL.

Kinetic monitoring of intracellular ATP content further corroborated these findings (Figure 1C,D). While control bacteria showed a progressive increase in ATP content over the initial 8 h of incubation, bacteria treated with ZnO NPs exhibited a dose-dependent suppression of ATP production as early as 2 h post-exposure, which remained stable up to 8 h. After a 24 h incubation, the ATP levels of bacteria treated with 100 μg/mL ZnO NPs returned to values comparable to those measured at baseline (time 0). In contrast, treatment with 250 and 500 µg/mL ZnO NPs resulted in further reductions in ATP content by 88 ± 0.07% and 59 ± 0.02%, respectively, compared to their initial baseline levels at time 0 (Figure 1D).

Collectively, these data demonstrate that ZnO NPs exert antibacterial activity against *S. sanguinis* by inhibiting bacterial growth, impairing metabolic activity, and suppressing ATP generation in a dose- and time-dependent manner.

### 2.2. Antimetabolic Effect of ZnO NPs

**Figure 2:**
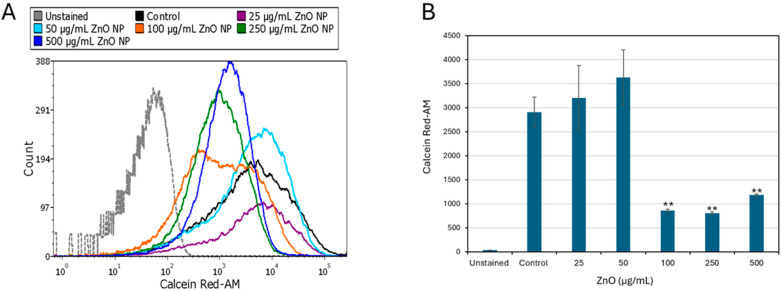
Decrease in metabolic activity measured by Calcein Red-AM fluorescence intensity, in *S. sanguinis* exposed to ZnO NPs. Calcein Red-AM fluorescence intensities were measured by flow cytometry after a 4 h exposure of the bacteria to the indicated concentrations of ZnO NPs. (**A**) Flow cytometric histograms of Calcein Red-AM fluorescence intensities and (**B**) the calculated geometric mean fluorescent intensity of the samples. Data are presented as mean ± SD of triplicates (*n* = 3). ** *p* < 0.001 compared to control.

To assess the early effects of ZnO NPs on *S. sanguinis* viability, Calcein Red-AM was used as a fluorogenic indicator of metabolic activity. The samples were analyzed by flow cytometry, which allowed the measurement of the metabolic activity of each individual bacterium within a bacterial population. Following a 4 h exposure, ZnO NPs induced a concentration-dependent decrease in fluorescence intensity (Figure 2), indicating impaired metabolic activity. While lower doses (25–50 μg/mL) did not show any significant antimetabolic effect, treatment with 100 μg/mL resulted in a significant decrease (70 ± 0.02% inhibition) in Calcein Red-AM fluorescence intensity (F(5, 12) = 6.98, *p* = 0.0028) as compared to control (Figure 2). These findings demonstrate that ZnO NPs suppress metabolic activity at early time points, with detectable effects occurring within 4 h of exposure.

### 2.3. Membrane Hyperpolarization and Loss of Membrane Integrity Are Late Events

**Figure 3:**
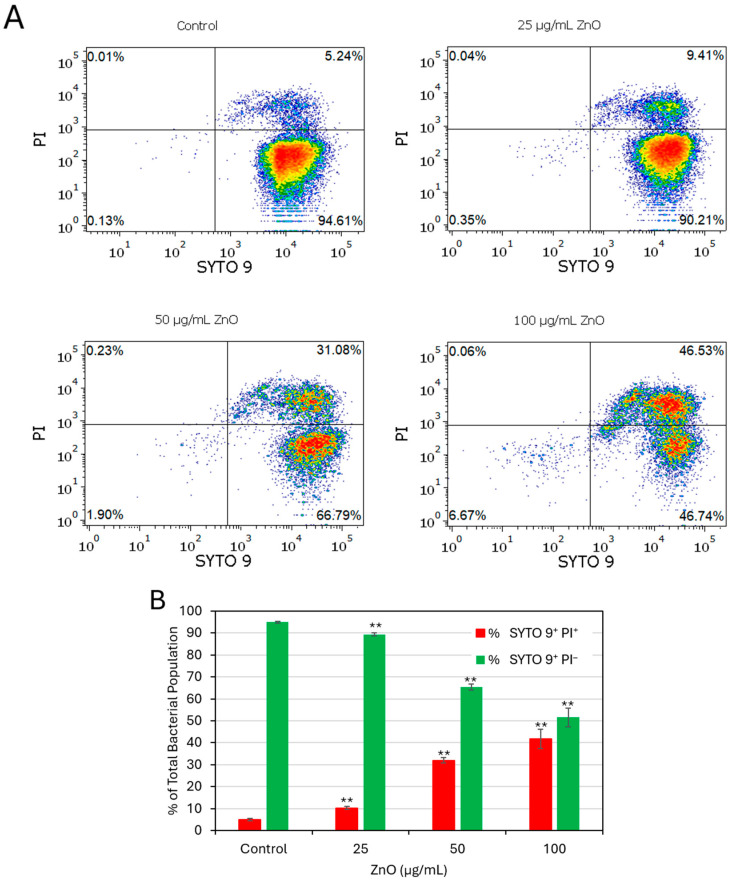
Live/dead SYTO 9/propidium iodide (PI) staining of planktonic-growing *S. sanguinis* after a 24 h exposure to ZnO NPs. Prolonged exposure to ZnO NPs increases membrane permeability. (**A**) PI vs. SYTO 9 dot plots of the different samples as indicated. (**B**) The percentages of SYTO 9^+^/PI^–^ live cells and SYTO 9^+^/PI^+^ dead cells. Data present mean ± SD of triplicates. ** *p* < 0.001 compared to control.

To determine whether ZnO NPs affected membrane integrity, the bacteria were stained with SYTO9/propidium iodide (PI) after 2 and 24 h of treatment. No significant PI uptake was observed after a 2 h exposure (Supplementary Figure S1A), whereas ZnO NPs caused a significant increase in PI-positive cells after a 24 h incubation (F(3, 8) = 176.03, *p* < 0.001) reaching 41 ± 4.3% at 100 µg/mL, and a decrease in live populations (F(3, 8) = 244.93, *p* < 0.001), indicating substantial membrane permeabilization after prolonged exposure (Figure 3A,B). Forward-scatter measurements (FSC-A) revealed additional ZnO NP-induced alterations in cell morphology after a 24 h incubation (Supplementary Figure S2), while no changes were detected after a short incubation of 2 h (Supplementary Figure S1B). FSC-A values increased significantly at concentrations above 100 µg/mL, suggesting that morphological changes had occurred.

**Figure 4:**
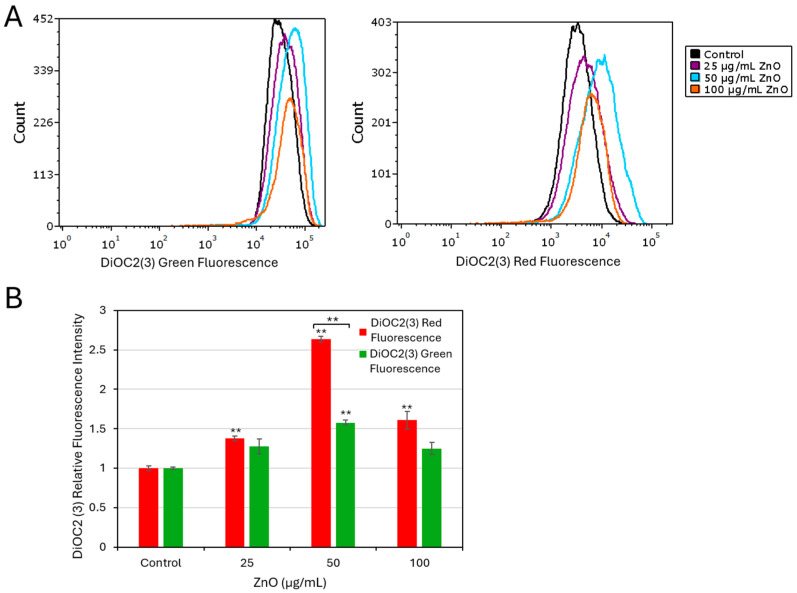
Prolonged exposure to ZnO NPs results in membrane hyperpolarization in *S. sanguinis*. Membrane potential was assessed using the DiOC2(3) potentiometric dye after a 24 h exposure of the bacteria to ZnO NPs. (**A**) Flow cytometric histograms of the green DiOC2(3) fluorescence intensities (FITC-A) and histograms of the red DiOC2(3) fluorescence intensities (PerCP-A). (**B**). The relative geometric mean of green and red emissions for control and ZnO NP-treated samples. A relative increase in red versus green fluorescence ratio is indicative of membrane hyperpolarization. Data present mean ± SD of triplicates. Brackets indicate significant differences between red and green channels (paired *t*-test). Significance levels: ** *p* < 0.001 in comparison to control.

Next, the bacterial membrane potential was assessed using the potentiometric dye DiOC2(3), which emits both green and red fluorescence. An increase in the red-to-green fluorescence intensity ratio is indicative of membrane hyperpolarization. Using this approach, ZnO NPs were found to induce only slight membrane hyperpolarization after a 2 h incubation (Supplementary Figure S1C), whereas significant variations for both green (*p* < 0.001) and red (*p* = 0.023) channels showed membrane hyperpolarization with 50 µg/mL ZnO NPs after a 24 h incubation (Figure 4A,B). Taken together, these findings suggest that the cellular metabolism is affected at an early stage following ZnO NPs treatment, whereas membrane permeabilization, morphological alterations, and loss of membrane potential occur later. This sequence of events is consistent with the progressive decline in bacterial viability observed over time (Figure 1C,D).

### 2.4. Effect of ZnO NPs on ROS Generation in S. sanguinis and the Influence of Alpha-Tocopherol on Bacterial Growth

**Figure 5:**
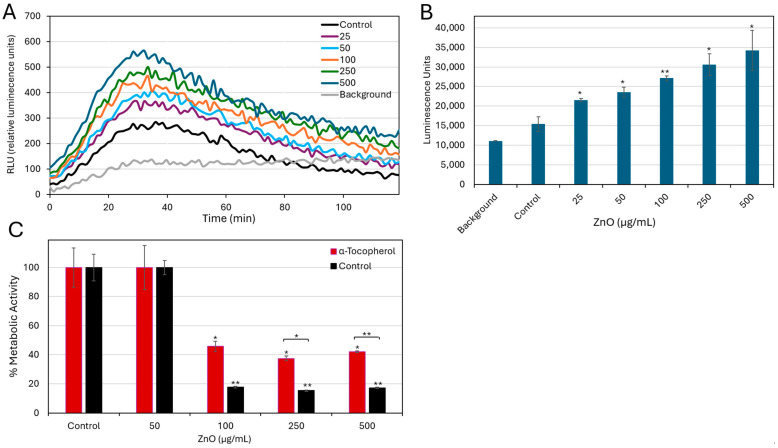
ZnO NPs induce ROS generation in a dose-dependent manner. (**A**) Real-time ROS production was monitored over a 2 h period following exposure to ZnO NPs at 25–500 µg/mL, as assessed by a luminescence-based luminol ROS assay. (**B**) Quantification of total ROS production during the 2 h exposure period, expressed as the area under the curve (AUC ± SD), was calculated from the data presented in panel A. The AUC values demonstrate a dose-dependent increase in ROS levels in response to ZnO NPs treatment. * *p* < 0.01, ** *p* < 0.001 compared to control. (**C**) Metabolic activity of planktonic growing bacteria measured by MTT metabolic assay after a 48 h exposure to ZnO NPs (50–500 µg/mL) in the absence or presence of α-tocopherol. Data represent mean ± SD of triplicates. Brackets indicate significant differences between α-tocopherol and control, determined by paired *t*-tests. * *p* < 0.0038, ** *p* < 0.001.

Previous studies have shown that ZnO NPs can induce oxidative stress, which is thought to contribute to their antibacterial action [12,15,16,17,18,19,20]. To determine whether ZnO NPs elicit oxidative stress in *S. sanguinis*, ROS generation was monitored over a 2 h period using a luminescence-based luminol assay [23]. As shown in Figure 5A,B, there is a clear dose-dependent increase in the relative ROS levels compared to the control group. The fold increase ranges from 2.42 ± 1.05-fold at the lowest dose (25 µg/mL) to 5.36 ± 2.6-fold at the highest dose (500 µg/mL) (F(5, 12) = 20.48, *p* < 0.001). To determine whether increased ROS production contributes to the antibacterial effect, the radical scavenger α-tocopherol was added to the bacteria in the absence or presence of increasing concentrations of ZnO NPs. α-Tocopherol is known to prevent lipid peroxidation [24,25,26], which is a common antibacterial mechanism for certain agents, including unsaturated fatty acids and ZnO NPs [18,25,27,28]. While α-tocopherol did not affect the metabolic activity of control bacteria, it partially attenuated the ZnO NP-mediated inhibition of metabolic activity (Figure 5C), increasing it from 18 ± 0.6% in the absence of α-tocopherol to 46 ± 3.6% in the presence of the scavenger. This partial rescue by α-tocopherol indicates that lipid peroxidation is involved in the antibacterial action, although it is not the sole mechanism.

### 2.5. Prolonged Exposure to ZnO NPs Leads to Morphological Changes in the Bacteria

**Figure 6:**
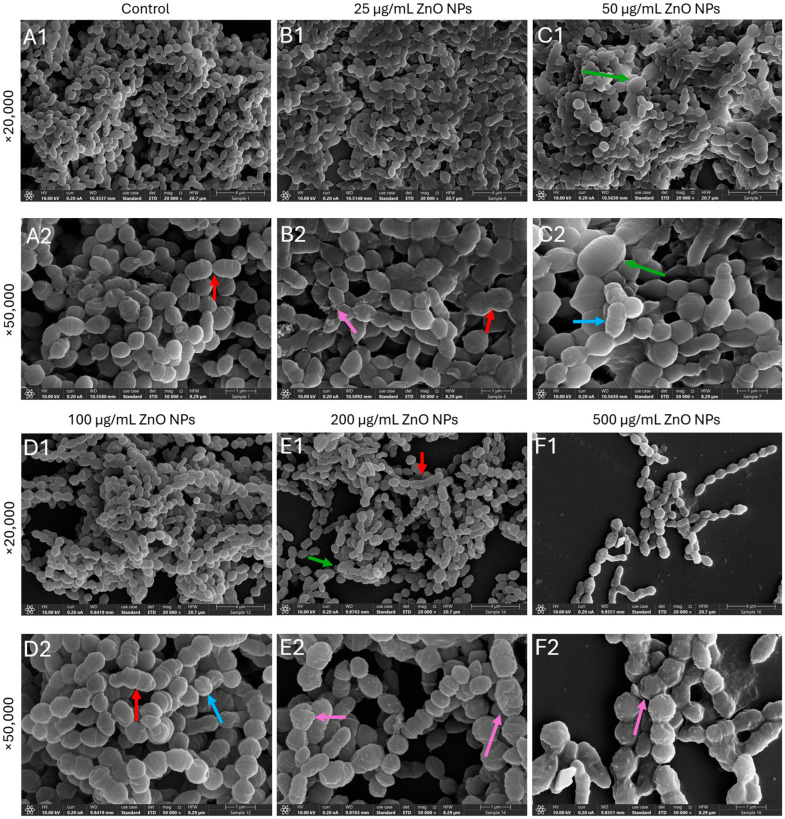
HR-SEM images of planktonic growing *S. sanguinis* following 24 h incubation with increasing concentrations of ZnO NPs. (**A1**,**A2**) Control, (**B1**,**B2**) 25 µg/mL ZnO NPs, (**C1**,**C2**) 50 µg/mL ZnO NPs, (**D1**,**D2**) 100 µg/mL ZnO NPs, (**E1**,**E2**) 200 µg/mL ZnO NPs, and (**F1**,**F2**) 500 µg/mL ZnO NPs. Images were acquired in a double-blind manner at 20,000× and 50,000× magnifications. Red arrows point to examples of membrane folding, representing less visible septa. Purple arrows point to examples of deposits. Blue arrows point to examples of surface roughening, and green arrows point to examples of enlarged bacteria.

While no significant morphological differences were detected after a 2 h exposure to ZnO NPs (Supplementary Figure S3), pronounced structural alterations were observed after 24 h (Figure 6). At ZnO NPs concentrations of 50 µg/mL and above, the bacteria appeared swollen and enlarged, and the membrane folding representing septa is less visible (Figure 6D1–F2). HR-SEM images captured at 20,000× and 50,000× magnification revealed irregular bacterial cell shapes, surface roughening, membrane deformation and collapse, and ZnO NPs adhering to the surfaces of damaged bacteria (Figure 6E1–F2). These ultrastructural changes observed at 24 h are consistent with the late onset of membrane permeabilization and depolarization detected by flow cytometry (Figure 3 and Figure 4).

**Figure 7:**
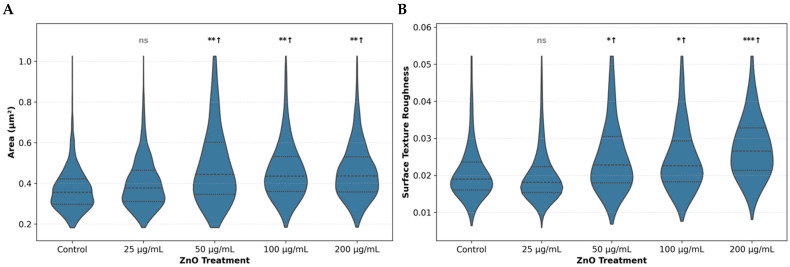
Computational quantitative assessment of ZnO NP-induced morphological alterations. Violin plots show the distribution of (**A**) bacterial projected cell area and (**B**) bacterial surface texture across ZnO NP treatments. The analysis was performed on six independent HR-SEM images per treatment (triplicate samples, 2 different fields per sample), yielding a total of 11,741 bacterial measurements. (**A**) Bacterial projected surface area increased significantly at concentrations ≥50 µg/mL. (**B**) Surface texture values increased significantly at 50 µg/mL ZnO NPs and higher concentrations. Significant differences are indicated as follows: ns = not significant; * = small (|δ| ≥ 0.147); ** = medium (|δ| ≥ 0.33); *** = large effect (|δ| ≥ 0.474) (Cliff’s delta). All comparisons were done relative to the control. Arrows indicate direction of change (↑ = increase). Horizontal lines within violins represent quartiles. Number of bacterial cells analyzed: control (*n* = 3030), 25 µg/mL (*n* = 2764), 50 µg/mL (n = 1861), 100 µg/mL (*n* = 2130), 200 µg/mL (*n* = 1956). Examples of the surface analysis in two HR-SEM images are provided in Supplementary Figure S5.

Computational cell area analysis demonstrated significant cellular enlargement at ZnO NPs concentrations ≥50 µg/mL (Figure 7A). Control bacteria had a mean cell area of 0.3703 ± 0.1067 µm^2^, while cells treated with 25 µg/mL (0.3997 ± 0.1245 µm^2^) showed no significant difference (δ = 0.133, ns). In contrast, bacteria exposed to 50, 100, and 200 µg/mL ZnO NPs exhibited significantly larger cell areas of 0.5153 ± 0.2562 µm^2^ (39.2% increase, δ = 0.393 **), 0.4645 ± 0.1554 µm^2^ (25.4% increase, δ = 0.411 **), and 0.4576 ± 0.1403 µm^2^ (23.6% increase, δ = 0.400 **), respectively (Figure 7A).

Computational quantification of bacterial surface texture revealed significant increases in surface roughness at ZnO NPs concentrations ≥50 µg/mL (Figure 7B). Control bacteria had a mean texture value of 0.0212 ± 0.0092, while treatment with 25 µg/mL (0.0203 ± 0.0088) showed no significant change (δ = −0.084, ns). However, exposure to 50, 100, or 200 µg/mL ZnO NPs for 24 h resulted in significantly rougher bacterial surfaces, with texture values of 0.0260 ± 0.0122 (22.7% increase, δ = 0.279 *), 0.0249 ± 0.0102 (17.6% increase, δ = 0.274 *), and 0.0279 ± 0.0099 (31.8% increase, δ = 0.491 ***), respectively.

These computational findings confirm that ZnO NPs induce significant increases in surface roughness and cell enlargement at concentrations ≥50 µg/mL, consistent with membrane disruption and cellular swelling observed in HR-SEM micrographs.

### 2.6. ZnO NPs Had an Antimetabolic Effect on Preformed, Mature Biofilms

**Figure 8:**
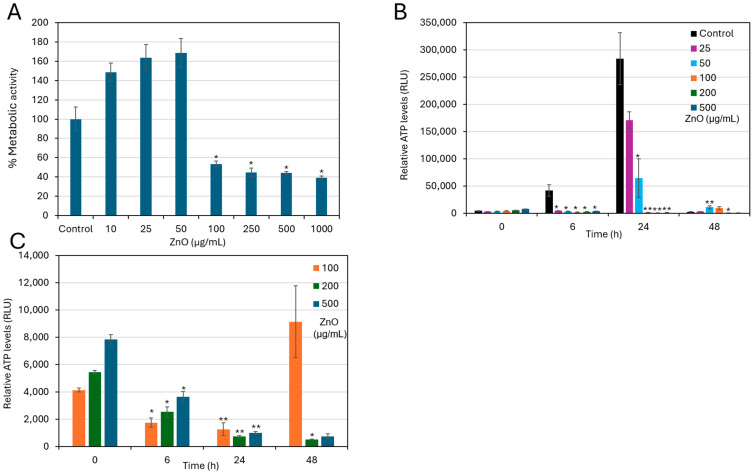
ZnO NPs exert an antimetabolic effect on established *S. sanguinis* biofilms. *S. sanguinis* were allowed to form biofilms for 48 h before being incubated with different concentrations of ZnO NPs for a further 48 h. Control biofilms received fresh medium without ZnO NPs. (**A**) Metabolic activity of established biofilms treated with ZnO NPs for 48 h as determined by MTT assay. The metabolic activity of control bacteria incubated for 48 h in medium without ZnO NPs was set to 100%. * *p* < 0.0071 compared to control. (**B**) Relative ATP levels in established *S. sanguinis* biofilms at various time points posttreatment as measured by the BacTiter Glo microbial viability assay. The ATP levels are expressed in relative luminescence units (RLU). (**C**) The Y-axis of panel B has been enlarged to better visualize the changes in ATP levels in ZnO NP-treated biofilms. Data are presented as mean ± SD of triplicates (*n* = 3). At each time point, the treated group was compared to its corresponding time-matched control using Bonferroni-corrected *t*-tests. * *p* < 0.01, ** *p* < 0.001.

Given that mature biofilms exhibit significantly greater resistance to antimicrobials than planktonic growing bacteria, primarily due to the protective extracellular polymeric substance (EPS) matrix [29], we also investigated the effects of ZnO NPs on biofilm-embedded bacteria. *S. sanguinis* biofilms were allowed to mature for 48 h before exposure to increasing concentrations of ZnO NPs for an additional 48 h. The metabolic activity of the resulting biofilms was then assessed using the MTT assay. As shown in Figure 8A, the metabolic activity of the preformed biofilms exposed to low concentrations of ZnO NPs (10–50 µg/mL) was similar to that of control biofilms. However, a marked decrease in metabolic activity was observed in biofilms exposed to 100 µg/mL and higher concentrations (F(7, 16) = 116,15, *p* < 0.001), indicating that ZnO NPs effectively impair the metabolic activity of mature biofilms in a dose-dependent manner (Figure 8A).

Consistent results were observed when measuring intracellular ATP levels of ZnO NP-treated biofilms using the BacTiter-Glo microbial viability assay. Control biofilms exhibited the expected increase in ATP levels in preformed biofilm at 6 h and 24 h following medium replenishment (Figure 8B), reflecting metabolic reactivation. However, treatment with 50 µg/mL ZnO NPs and higher concentrations prevented this increase in intracellular ATP levels (Figure 8B), and even a progressive depletion of ATP was observed over time with 100 µg/mL and higher ZnO NPs concentrations (Figure 8C), confirming antimetabolic effects.

While lower concentrations (25–50 µg/mL) caused a delayed increase in ATP at 6 and 24 h, the ATP levels in both control and low-dose treatments (25–50 µg/mL) returned to approximately their baseline levels (time 0) by 48 h, when the bacteria entered a sessile state. In contrast, at concentrations ≥ 100 µg/mL, intracellular ATP levels declined below the initial baseline (time 0) (Figure 8C). After 48 h, biofilms treated with 200 or 500 µg/mL retained only 10 ± 2% residual metabolic activity relative to time 0 (Figure 8C). Collectively, these findings demonstrate that ZnO NPs rapidly suppress the metabolic activity of established *S. sanguinis* biofilms and effectively halt their further development in a concentration-dependent manner.

### 2.7. HR-SEM Imaging of Established S. sanguinis Biofilms Treated with ZnO NPs

**Figure 9:**
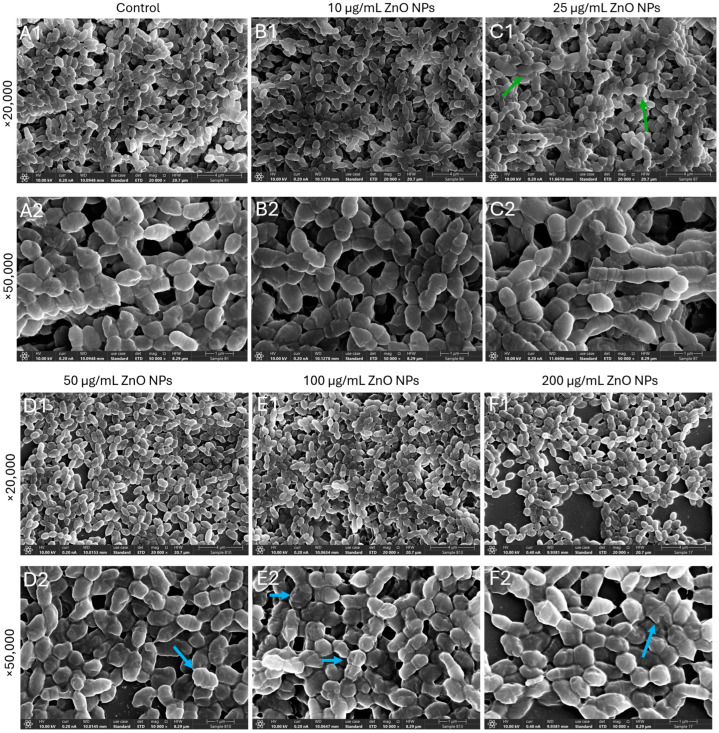
HR-SEM images of 48 h established biofilms of *S. sanguinis* after incubation with increasing concentrations of ZnO NPs for another 48 h. (**A1**,**A2**) Control, (**B1**,**B2**) 10 µg/mL ZnO NPs, (**C1**,**C2**) 25 µg/mL ZnO NPs, (**D1**,**D2**) 50 µg/mL ZnO NPs, (**E1**,**E2**) 100 µg/mL ZnO NPs, and (**F1**,**F2**) 200 µg/mL ZnO NPs. Images were acquired blindly at 20,000× and 50,000× magnifications. Green arrows point to enlarged bacteria, and blue arrows point to surface roughening.

HR-SEM imaging was used to examine the structural impact of ZnO NPs on mature *S. sanguinis* biofilms that had been allowed to form for 48 h before treatment with ZnO NPs for a further 48 h. As shown in Figure 9A1,A2, untreated biofilms exhibited a dense, continuous architecture composed of tightly packed bacterial clusters. Exposure of mature biofilms to 10 µg/mL ZnO NPs for 48 h did not markedly alter the overall biofilm organization (Figure 9B1,B2). The biomass remained compact, and bacterial cells largely maintained their classical ovoid morphology with regularly folded surface structures, with only minor loosening of the upper biofilm layers. At 25 and 50 µg/mL ZnO NPs, subtle morphological alterations became apparent (Figure 9C1–D2), with some bacteria displaying swelling similar to that observed in planktonic cells exposed to ZnO NPs (Figure 6A1,A2). At higher ZnO NPs concentrations (100–200 µg/mL) (Figure 9E1–F2), pronounced structural disruption was observed on the bacterial surface. The bacteria showed irregular contours and surface alterations consistent with membrane stress or deformation. Altogether, these observations indicate that ZnO NPs disrupt the integrity and architecture of preformed *S. sanguinis* biofilms in a concentration-dependent manner.

## 3. Discussion

The development of bioactive materials that selectively target pathogenic oral bacteria while preserving the ecological balance of commensal populations is a key objective in modern preventive dentistry [15]. Although our previous research elucidated the mechanisms by which zinc oxide nanoparticles (ZnO NPs) inhibit the cariogenic pathogen *S. mutans*, assessing their effects on oral commensal species such as *S. sanguinis* is essential for understanding their broader impact on dental biofilm homeostasis [9,21]. As a primary colonizer of the tooth surface, *S. sanguinis* facilitates the subsequent attachment of later colonizers in dental plaque development. [30]. Therefore, the ability of ZnO NPs to disrupt this biofilm foundation has broad implications for controlling the development of pathogenic multispecies biofilms [4,9].

Our findings demonstrate that *S. sanguinis* is significantly more sensitive to ZnO NPs than the cariogenic *S. mutans* [21]. The MIC for *S. sanguinis* was determined to be 100 μg/mL, a five-fold lower dose than the 500 μg/mL required for *S. mutans* [21]. This species-specific sensitivity is further highlighted by comparing metabolic responses. While *S. mutans* required 500 μg/mL to achieve significant growth inhibition and ATP depletion [21], *S. sanguinis* exhibited comparable suppression at a five-fold lower concentration of 100 μg/mL. These dissimilarities highlight differences in susceptibility of a commensal species relative to a pathogen and raise concerns that indiscriminate application of ZnO NPs could inadvertently promote dysbiosis by preferentially suppressing protective bacteria rather than cariogenic species [4,9,30]. This vulnerability is supported by the fact that pioneer colonizers such as *S. sanguinis* often lack complex extracellular polymeric substances (EPS) during the initial stages of attachment [31], which distinguishes them from later-stage or more adapted pathogens, such as *S. mutans,* which are robust EPS producers [32]. Structurally, the matrix is composed of polysaccharides, proteins, lipids, and extracellular DNA, which collectively affect biofilm formation and fluid dynamics. The biofilm matrix functions as both a physical and chemical barrier, which significantly retards the diffusion of toxic and charged molecules into the biofilm [32,33]. Early colonizers or bacteria not yet embedded within the EPS lack this protective matrix, making them highly vulnerable to antimicrobial agents.

The smaller size of nanoparticles (5–30 nm) allows for a higher surface-to-volume ratio [11,12], which facilitates closer interaction with the bacterial membrane and triggers localized damage through the generation of reactive oxygen species (ROS) by the bacteria and the release of toxic Zn^2+^ ions from the NPs [34].

A key finding of this study is the temporal separation between metabolic impairment and physical membrane damage of *S. sanguinis* in the presence of ZnO NPs. The primary driving force of growth inhibition in *S. sanguinis* appears to be early metabolic suppression, characterized by a significant reduction in ATP production and esterase activity, detectable as early as 2 h after ZnO NPs exposure. At this time point, bacterial membrane integrity is preserved, and bacterial morphology remains mostly indistinguishable from that of untreated controls. A notable exception was observed at 100 µg/mL ZnO NPs, where a reduction in bacterial surface folding was noted (Supplementary Figure S3E). This morphological alteration suggests potential interference with septum formation, which may be due to an inhibition of cell division. Substantial changes in membrane properties were observed after a 24 h exposure to ZnO NPs, including increased membrane permeabilization as indicated by propidium iodide (PI) uptake reaching 41 ± 4.3% at 100 µg/mL, and pronounced membrane hyperpolarization.

Altogether, these findings indicate that ZnO NPs initially suppress essential metabolic processes, with overt membrane damage emerging later as a secondary event. A similar sequence of events has been reported by Tiwari et al. [18], who showed that ROS production and lipid peroxidation precede membrane rupture and leakage of cellular contents (reducing sugars, proteins, DNA) in *Acinetobacter baumannii*, as confirmed by TEM imaging of disrupted envelopes after ZnO NPs treatment.

The late-onset membrane hyperpolarization observed in *S. sanguinis* can be explained by two mechanistic pathways [35]. First, H^+^-dependent efflux systems serve as homeostatic regulators. Changes in proton coupling can lead to a more negative membrane potential [36]. Initial membrane hyperpolarization may occur when the bacteria activate proton pumps to expel toxic Zn^2+^ ions or counteract ROS-induced proton leaks, leading to a temporary increase in membrane potential [37]. Second, during metabolic collapse, the F_1_F_o_-ATPase can operate in reverse, hydrolyzing residual ATP to actively pump protons outward. ZnO-driven membrane hyperpolarization can overstimulate the proton motive force (PMF)-dependent ATP synthase in reverse mode [38]. This process can generate an abnormally negative membrane potential, often associated with declining metabolic activity and disrupted ion flux [39]. In our study, ATP levels dropped rapidly within 2–4 h, while hyperpolarization and membrane damage appeared only after 24 h. This sequence suggests that hyperpolarization results from metabolic collapse rather than an early compensatory ion efflux.

The late-stage bacterial injury was further corroborated by high-resolution scanning electron microscopy (HR-SEM) and computational morphometric analysis. After 24 h of exposure to ZnO NPs concentrations ≥50 μg/mL, *S. sanguinis* cells appeared markedly swollen and enlarged, suggesting that the bacteria had undergone osmotic stress. Quantitative analysis revealed a significant, dose-dependent increase in surface roughness, reflecting membrane deformation. The observation of aggregates adhering directly to damaged bacterial surfaces suggests physical contact between ZnO NPs and the cell wall, which contributes to the eventual mechanical failure of the membrane [12,17].

Beyond the biological findings, this work underscores the value of computational morphometric analysis as an objective tool for quantifying nanoparticle-induced bacterial damage. The combination of deep-learning-based segmentation with geometric and surface-texture measurements enabled the detection of subtle, dose-dependent morphological changes that are not readily discerned by qualitative microscopy alone. By converting high-resolution SEM images into reproducible numerical descriptors, this approach provides a robust framework for linking structural perturbations to bacterial physiological states. It supports comparative analysis of antimicrobial nanomaterials across experimental conditions.

The generation of reactive oxygen species (ROS) is often suggested as a key mechanism behind ZnO NP-mediated cytotoxicity [10,15]. We observed a dose-dependent increase in ROS production, reaching a level of 5.36 ± 2.6-fold higher with 500 µg/mL ZnO NPs in comparison to control bacteria. Co-treatment with the radical scavenger α-tocopherol partly rescued the antibacterial effect of ZnO NPs, suggesting that lipid peroxidation contributes to the antibacterial effect, although it is not the sole mechanism. α-Tocopherol failed to completely restore bacterial viability and metabolic activity to the level of control bacteria. Consistent with a previous report [17], direct interactions between the nanoparticles and the bacteria, together with the contribution of released Zn^2+^ ions, appear to play more prominent roles in the antibacterial activity. We cannot exclude the possibility that the presence of lipophilic α-tocopherol may interfere with the interaction of ZnO NPs with the bacterial membrane.

Importantly, our study shows that ZnO NPs are effective not only against planktonic cells but also against mature, 48 h-old *S. sanguinis* biofilms. ZnO NPs at ≥100 μg/mL effectively impaired the metabolic activity and the structural architecture of the immobilized bacteria. The rapid suppression of ATP levels within established biofilms suggests that ZnO NPs can penetrate the biofilm matrix to reach deeply embedded cells, thereby overcoming diffusion barriers typically present in mature biofilms [10,16].

Despite the mechanistic insights, this study has several limitations that should be acknowledged. Although *S. sanguinis* is recognized as an important early colonizer, this in vitro investigation was conducted using a single-species model, which does not fully capture the dynamic interactions and structural complexity of polymicrobial oral biofilms encountered in clinical settings. In vivo, biofilm development involves intricate interspecies communication, spatial organization, and metabolic cooperation that may influence susceptibility to ZnO NPs. Furthermore, this study evaluated a defined range of ZnO NP concentrations. The long-term ecological impact of varying clinical dosages of ZnO NPs on the oral microbiome, including potential shifts in microbial composition and resilience, remains to be determined. Future research should therefore incorporate multispecies biofilm models that better simulate the oral ecosystem. Moreover, the development of targeted delivery systems or controlled-release formulations for ZnO NPs warrants further exploration. Such approaches are essential to ensure that the potent antibacterial efficacy of these nanoparticles can be harnessed to eradicate pathogens without permanently disrupting the commensal homeostasis necessary for long-term oral health.

## 4. Materials and Methods

### 4.1. Materials

Zinc oxide (ZnO) nanoparticles (<100 nm) supplied as a 20% aqueous suspension were obtained from Sigma Aldrich (Cat. No. 721077; St. Louis, MO, USA). According to the Certificate of Analysis, the average particle size is ≤40 nm. The physicochemical characterization of these ZnO NPs has been reported previously [21].

### 4.2. Bacterial Cultivation of Streptococcus sanguinis

*Streptococcus sanguinis* (NCTC7863) was used as the bacterial model strain in this study. The bacterial species was confirmed by matrix-assisted laser desorption/ionization time-of-flight mass spectrometry (MALDI-TOF MS) (bioMérieux, Marcy l’Etoile, France) at the Department of Clinical Microbiology and Infectious Diseases, Hadassah-Hebrew University Medical Center (Jerusalem, Israel), and by sequencing the 16S rRNA PCR product generated by the primer pairs F: 5′-AGTTTGATCCTGGCTCAG-3′ and R: 5′-GGTTACCTTGTTACGACTT-3′ [40] using the Oxford Nanopore sequencing service provided by Plasmidsaurus (Cologne, Germany). Bacterial DNA was extracted with the NucleoSpin Microbial DNA isolation kit (Machery-Nagel GmbH & Co., Düren, Germany). Microscopically, the bacteria appeared as ovoid shapes in long chains. The bacteria were cultured in Wilkins–Chalgren broth (Oxoid, Basingstoke, UK). A starter culture was prepared by inoculating 1 mL of a frozen stock into 35 mL of Wilkins–Chalgren broth, followed by incubation for 48 h at 37 °C under anaerobic conditions using AnaeroGen sachets (Thermo Scientific, Oxoid, UK) in hermetically sealed jars [41]. Then, 1 mL of this bacterial culture was transferred to 35 mL of Wilkins–Chalgren broth for another 48 h of incubation under anaerobic conditions. The bacteria were then centrifuged at 10,000× *g* for 5 min, resuspended in fresh Wilkins–Chalgren broth, and used for the experiments. All procedures were performed under these incubation conditions.

### 4.3. Planktonic Culture of S. sanguinis and MTT Metabolic Assay

To evaluate the effect of ZnO NPs on *S. sanguinis*, the bacterial starter culture was centrifuged at 10,000× *g* for 5 min, resuspended in fresh Wilkins–Chalgren broth, and adjusted to an OD_600nm_ of 0.2. Then, 100 µL of this bacterial suspension was dispensed into flat-bottom 96-well tissue culture plates (Corning, Kennebunk, ME, USA), mixed with 100 µL of two-fold serial dilutions of ZnO NPs prepared in Wilkins–Chalgren broth, resulting in a final initial bacterial OD_600nm_ of 0.1. The plates were incubated anaerobically at 37 °C for 48 h. After incubation, absorbance was measured at 600 nm using a Multiskan SkyHigh microplate reader (Thermo Scientific Multiskan SkyHigh, Life Technologies, Holdings Pte Ltd., Singapore) to assess bacterial growth. The MIC was defined as the lowest concentration resulting in no visible bacterial growth. The percentage growth inhibition of treated bacteria was calculated in comparison to control bacteria at the same time point after subtracting the background. Subsequently, bacterial metabolic activity of the planktonic growing bacteria was assessed by adding 50 µL of a 5 mg/mL MTT solution (GoldBio, St. Louis, MO, USA) in PBS directly to the bacterial broth, followed by a 4 h incubation under anaerobic conditions at 37 °C [42]. The absorbance at 570 nm was then measured in a microplate reader.

### 4.4. Determination of Intracellular ATP Content Using BacTiter-Glo Microbial Cell Viability Assay

Planktonic bacterial viability was further measured using the BacTiter-Glo Microbial Cell Viability Assay (Promega, Madison, WI, USA), which detects ATP as an indicator of metabolically active cells [43]. At specified time points, 100 µL samples were taken from bacterial cultures grown in 15 mL medium with an initial OD_600nm_ of 0.1 and transferred into µ-clear 96-well white flat-bottom microplates (Greiner Bio-One GmbH, Frickenhausen, Germany). An equal volume (100 µL) of BacTiter-Glo reagent was added to each well. Medium without bacteria, with or without ZnO NPs, served to measure background luminescence, and ZnO NPs were confirmed not to interfere with the assay. After a 10 min incubation at room temperature, luminescence was measured using an Infinite M200 plate reader (Tecan Group Ltd., Männedorf, Switzerland).

### 4.5. ATP Content and MTT Metabolic Activity of Preformed Biofilms

*S. sanguinis* biofilm was allowed to form for 48 h in a 24-well plate by incubating the bacteria in 2 mL of Wilkins–Chalgren broth. The medium was then replaced with 2 mL of fresh Wilkins–Chalgren broth containing different concentrations of ZnO NPs, followed by another 48 h of incubation without changing the medium. At the end of incubation, the supernatant was discarded, and bacterial metabolic activity in the biofilms was assessed by adding either 0.5 mg/mL MTT for a 4 h incubation at 37 °C as described in Section 4.3 or adding 100 μL of the BacTiter-Glo Microbial Cell Viability reagent as described in Section 4.4.

### 4.6. Assessment of Metabolic Activity by Flow Cytometry Using Calcein Red-AM

Calcein Red-AM (Biolegend, San Diego, CA, USA) was used as a fluorogenic indicator for metabolic activity [44]. 50 µg of Calcein Red-AM were dissolved in 50 µL DMSO to prepare a 1 mM stock solution, which was then diluted 1:200 in Wilkins–Chalgren broth to get a 5 µM working solution. *S. sanguinis* cultures were adjusted to an initial OD_600nm_ of 0.3, centrifuged for 5 min at 5000× *g*, and resuspended in Wilkins–Chalgren broth containing ZnO NPs at concentrations ranging from 0 to 500 µg/mL. Bacteria were incubated with ZnO NPs for 4 h under anaerobic conditions, then Calcein Red-AM was added to reach a final concentration of 5 µM and the samples were incubated for 1 h at 37 °C. After incubation, the bacteria were washed, resuspended in 1 mL PBS, and analyzed by flow cytometry (LSR-Fortessa, BD Biosciences) using DsRed channel settings (Ex 561 nm/Em 574 nm). The fluorescence intensity correlates with intracellular esterase activity, reflecting the bacteria’s metabolic activity. Data are expressed as the geometric mean fluorescence intensity, the Nth root of all observed values. Fifty thousand events were collected for each sample.

### 4.7. SYTO 9/PI Live/Dead Staining

After a 2 h or 24 h exposure of *S. sanguinis* (initial OD_600nm_ = 0.3) to ZnO NPs, the bacteria were centrifuged and stained with 1 µM SYTO 9 (Molecular Probes, Life Technologies, Carlsbad, CA, USA) and 2 µg/mL propidium iodide (PI) (Sigma, St. Louis, MO, USA) in 1 mL PBS for 20 min. Green and red fluorescence were then measured by flow cytometry (LSR Fortessa, BD Biosciences, San Jose, CA, USA) using excitation/emission settings of 488/520 nm for SYTO 9 and 561/586 nm for PI, respectively [45]. Each sample included 50,000 events, performed in triplicate. SYTO 9 stains the nucleic acids of both live and dead cells, emitting green fluorescence, while PI enters only cells with compromised membranes and emits red fluorescence upon nucleic acid binding. Cells that have lost their nucleic acid content due to cytoplasmic leakage appear as SYTO 9-low or negative and PI-negative populations.

### 4.8. Membrane Potential Determination by Flow Cytometry

The effect of ZnO NPs on the membrane potential of *S. sanguinis* was studied using the BacLight Membrane Potential Kit (Molecular Probes, Life Technologies, Eugene, OR, USA), following the manufacturer’s instructions [45]. A Wilkins–Chalgren culture adjusted to an OD_600nm_ of 0.3 was exposed to various concentrations of ZnO NPs for 2 h or 24 h. At the end of incubation, cells were centrifuged, resuspended in 1 mL PBS, and stained with the potentiometric dye 3,3′-diethyloxacarbocyanine iodide (DiOC2(3)) at a final concentration of 30 µM. Samples were incubated for 20 min at room temperature before flow cytometric analysis. The fluorescence intensity was measured by flow cytometry (LSR-Fortessa, BD Biosciences) using 488 nm excitation with green (530 nm) and red (610/620 nm) emission filters, and 50,000 events were collected per sample. Data were acquired using BD FACSDiva software 8.0.1 and analyzed with FCS Express 7.12.0007 De Novo Software. For each condition, the geometric mean fluorescence intensities of both green and red fluorescence were calculated. Values were normalized to the untreated control, which was set to 1. Membrane potential changes were assessed by calculating the red/green fluorescence ratio; an increase in the ratio indicated membrane hyperpolarization.

For statistical analysis, FITC-A and PerCP-A channels were analyzed using separate one-way ANOVAs. To account for multiple comparisons (effect of dose and red vs. green divergence), a global Bonferroni-corrected threshold of *p* < 0.005 was applied. Differences between fluorescence channels at each concentration were evaluated using paired *t*-tests.

### 4.9. Reactive Oxygen Species (ROS) Production

Reactive oxygen species (ROS) production by *S. sanguinis* was quantified using a luminol-based chemiluminescence assay [21]. An overnight culture of *S. sanguinis* adjusted to an OD_600nm_ of 0.2 in Hank’s Balanced Salt Solution (HBSS; without phenol red) supplemented with D-glucose to a final concentration of 1%. In a μ-clear white 96-well plate (Corning, Kennebunk, ME, USA), 100 µL of the bacterial suspension was mixed with 100 µL of ZnO NPs suspension prepared in HBSS, yielding final ZnO NPs concentrations between 0–0.5 mg/mL. Luminol (50 µM final; prepared from a 50 mM DMSO stock) and horseradish peroxidase (HRP; 4 U/mL; Sigma St. Louis, MO, USA) were added to all wells. Bacteria-free controls with and without ZnO NPs were included to determine background chemiluminescence. Chemiluminescence was measured using an Infinite M200 plate reader (Tecan) with a 1 s integration time per well, recorded every 74 s for up to 2 h. ROS production was expressed as relative luminescence units (RLU) over time to monitor kinetic changes.

### 4.10. ROS Scavenging Assay with α-Tocopherol

To determine whether the antibacterial activity of ZnO NPs was mediated by the generation of reactive oxygen species (ROS), a rescue experiment was performed using α-tocopherol (Sigma-Aldrich, St. Louis, MO, USA), which is a potent antioxidant agent [24]. A 10 mM stock solution of α-tocopherol was prepared in absolute ethanol and further diluted in Wilkins–Chalgren broth to a working concentration of 100 μM.

Overnight cultures of *S. sanguinis* were adjusted to an OD_600nm_ of 0.3 and treated with ZnO NPs at concentrations ranging from 0 to 500 µg/mL in the presence or absence of 100 µM α-tocopherol. To account for potential solvent effects, control groups were treated with an equivalent volume of 1% ethanol (the vehicle for α-tocopherol). The cultures were incubated under anaerobic conditions as described above. Bacterial growth and metabolic activity were monitored to assess if the addition of the antioxidant could mitigate the antibacterial effects of ZnO NPs.

### 4.11. Morphological Imaging by High-Resolution Scanning Electron Microscopy (HR-SEM)

To visualize the morphology of planktonically growing cells after 2 h and 24 h exposure to ZnO NPs, *S. sanguinis* cultures with an initial OD_600nm_ of 0.3 were rinsed once with double-distilled water (DDW) and fixed in 4% glutaraldehyde (Electron Microscopy Sciences, Hatfield, PA, USA) in DDW for 2 h [46]. The fixed cells were then washed once with DDW and resuspended in 50 µL DDW. Aliquots of 10 μL were then applied to 0.7 × 0.7 cm glass pieces prepared from standard microscope slides and allowed to air-dry. Specimens were subsequently sputter-coated with iridium and imaged using an Analytical High-Resolution Scanning Electron Microscope (Apreo 2S LoVac, Thermo Fisher Scientific, Waltham, MA, USA) at various magnifications.

For HR-SEM images of preformed biofilm, the bacteria (initial OD_600nm_ of 0.1) in 2 mL broth were allowed to form biofilms on 0.7 × 0.7 cm glass pieces for 48 h in 24-well plates, and then exposed for another 48 h to increasing concentrations of ZnO NPs in fresh broth. At the end of incubation, the glass pieces were washed twice with DDW, then fixed in 4% glutaraldehyde (Electron Microscopy Sciences, Hatfield, PA, USA) in DDW for 2 h, then washed in DDW and allowed to dry.

### 4.12. Computational Analysis of Bacterial Morphology

Quantitative morphological analysis was performed on HR-SEM images using Python programming language (Python 3.10, Python Software Foundation, https://www.python.org/, URL accessed on 28 September 2025) for all computational analyses and figure preparation. Six SEM images per treatment condition at ×20,000 magnification were cropped to remove scale bars and rescaled to the intensity range [0, 255]. A fixed pixel-to-micrometer conversion factor was applied uniformly across all images for consistent processing across all treatment conditions. Identical computational parameters and thresholds were applied to all images, ensuring that any systematic measurement bias would affect all treatment groups equally, thereby preserving the validity of between-group comparisons.

#### 4.12.1. Computational Bacterial Segmentation and Morphological Analysis

Bacterial segmentation was performed on HR-SEM images using the Cellpose deep-learning model [47] (https://github.com/MouseLand/cellpose, URL accessed on 28 September 2025) and the pre-trained models provided with the software package. The reliability and applicability of this approach were verified in a series of HR-SEM images taken from the same samples in different contexts. Thereafter, the pre-trained model was used without further fine-tuning or custom training, because it demonstrated sufficient accuracy in identifying individual *S. sanguinis* cells (Supplementary Figure S5). Each segmented cell was fitted to an ellipse using OpenCV’s least-squares method, from which geometric properties, including centroid coordinates, major and minor axis lengths, area, and orientation angle, were computed using scikit-image region props (https://scikit-image.org/) and OpenCV-Python (https://pypi.org/project/opencv-python/) modules (URL accessed on 28 September 2025). Bacteria with fewer than five contour points or an area below 1000 square pixels (0.183 μm^2^), which represent segmentation artifacts, were excluded to ensure reliable measurements.

#### 4.12.2. Background Filtering

A local size-based filtering approach was applied to remove hidden or background bacteria, excluding those with an area <0.7 × the mean neighbor area to avoid overlap with neighbors. This filtering removed 20–32% of initially segmented objects across treatment conditions. Neighbor detection was performed using a KD-Tree spatial index (https://docs.scipy.org/doc/scipy/reference/generated/scipy.spatial.cKDTree.html, URL accessed on 28 September 2025) to efficiently identify adjacent bacteria based on minimum perimeter-to-perimeter distance (<5 pixels, 0.068 μm). The perimeter distance was calculated by generating 100 equally spaced points along the perimeter of the ellipse representing each bacterium and computing the minimum Euclidean distance between all pairs of points. This approach removes bacteria obscured by other bacteria (therefore, smaller) and background artifacts.

#### 4.12.3. Surface Texture Analysis

Surface texture was quantified to assess bacterial membrane damage using polynomial surface modeling of grayscale intensity variations. For each segmented bacterium, the binary mask was eroded (7 × 7 kernel, 2 iterations) to exclude edge artifacts. A second-order polynomial surface $z=a_{0}+a_{1}x+a_{2}y+a_{3}x^{2}+a_{4}y^{2}+a_{5}xy$ was then fitted to the normalized intensity values (range [0, 1]) within the eroded region using least-squares regression. The metric quantifies local intensity deviation from the smooth, fitted surface, where the polynomial model removes global intensity gradients (due to specimen tilt or technical intensity gradients) and gentle bacterial curvature, thereby isolating high-frequency texture variations attributable to surface morphology. An example of the polynomial surface-fitting procedure for a smooth (control) and a rough (ZnO NP-treated) bacterium is shown in Supplementary Figure S4. Due to image normalization [0, 1] and polynomial surface removal, texture values span a small numerical range (0.016–0.033 for the analyzed bacteria), where higher values indicate rougher, damaged surfaces and lower values indicate smoother, intact surfaces. This computational approach provides a novel, objective, quantitative assessment of structural alterations across treatment conditions, enabling accurate statistical comparisons to identify significant changes induced by the treatments.

#### 4.12.4. Statistical Analysis of Computational Parameters

Group comparisons were performed using Cliff’s delta (δ), a non-parametric effect size measure. Effect sizes were categorized as large (|δ| ≥ 0.474), medium (|δ| ≥ 0.33), small (|δ| ≥ 0.147), or not significant (|δ| < 0.147), following established guidelines [48].

### 4.13. Statistical Analysis of the Biological Experiments

The experiments were performed in triplicate and repeated independently three times. The data are presented as the average ± standard deviation. Statistical significance between groups was assessed using the one-way analysis of variance (ANOVA). When a significant main effect was observed (*p* < 0.05), post hoc pairwise comparisons were conducted using independent *t*-tests with a Bonferroni correction to account for multiple comparisons. The significance threshold for each figure was adjusted by dividing the alpha level (0.05) by the number of planned comparisons specific to that dataset (e.g., *p* < 0.0071 for 7 comparisons, *p* < 0.0083 for 6 comparisons, or *p* < 0.01 for 5 comparisons). For experiments involving internal comparisons within the same sample, such as red vs. green fluorescence or antioxidant interactions, paired *t*-tests were utilized following the ANOVA to ensure appropriate analysis of dependent variables. Significance levels are indicated in the figure legends as * *p* < adjusted threshold and ** *p* < 0.001.

## 5. Conclusions

In conclusion, ZnO NPs exhibit pronounced antimicrobial and antibiofilm activity against *S. sanguinis* through a defined temporal sequence characterized by early metabolic suppression, followed by membrane hyperpolarization and structural disintegration. The antibacterial process appears to be partially influenced by ROS, which contributes to toxicity, but does not represent the sole mechanism of action. These findings underscore the potential application of ZnO NPs as bioactive components in dental restorative materials. Future formulation strategies should focus on controlled drug release and/or targeted delivery approaches to balance effective pathogen eradication with preservation of the commensal microbiota, which is essential for long-term oral health.

## Data Availability

All relevant data are presented in the manuscript. Raw data for the figures are available upon reasonable request from the corresponding author.

## Supplementary Materials

The following supporting information can be downloaded at https://www.mdpi.com/article/10.3390/ijms27062782/s1.

## Abbreviations

The following abbreviations are used in this manuscript:

ATP	Intracellular adenosine triphosphate
AUC	Area under the curve
EPS	Extracellular polysaccharide substance
DiOC2(3)	3,3′-diethyloxacarbocyanine iodide
FSC-A	Forward-scatter measurements
HRP	Horseradish peroxidase
HR-SEM	High-resolution scanning electron microscopy
MTT	3-(4,5-dimethylthiazol-2-yl)-2,5-diphenyltetrazolium bromide
PI	Propidium iodide
RLU	Relative luminescence units
ROS	Reactive oxygen species
*S. mutans*	*Streptococcus mutans*
*S. sanguinis*	*Streptococcus sanguinis*
TEM	Transmission electron microscopy
ZnO NPs	Zinc oxide nanoparticles
