# Supplementary material for: Susceptibility of the Oral Commensal Bacterium Streptococcus sanguinis to ZnO Nanoparticles
Source: Int J Mol Sci. 2026 Mar 19;27(6):2782. doi: 10.3390/ijms27062782 (PMC13027026; doi:10.3390/ijms27062782)
Supplement: Supplementary file 1 [file ijms-27-02782-s001.zip › ijms-4178609-supplementary.pdf]

Susceptibility of the Oral Commensal Bacterium *Streptococcus sanguinis* to ZnO Nanoparticles

Raphaelle Emram [1,2], Ronit Vogt Sionov [1], Adi Aharoni [1,3,4], Sarah Gingichashvili [1,3], Noa E. Cohen [3], Vitaly Gutkin [5], Moshe Amitay [4], Asaf Wilensky [2], Doron Steinberg [1,†] and Rawi Assad [2,*,†]

1 Faculty of Dental Medicine, Institute of Biomedical and Oral Research (IBOR), The Hebrew University of Jerusalem, Ein Kerem Campus, Jerusalem 9112102, Israel; raphaell.emram@mail.huji.ac.il (R.E.); ronit.sionov@mail.huji.ac.il (R.V.S.); adi.ahaorni@mail.huji.ac.il (A.A.); sophiko.gingichashvili@mail.huji.ac.il (S.G.); dorons@ekmd.huji.ac.il (D.S.)
2 Department of Periodontology, Hadassah Medical Center, Faculty of Dental Medicine, Hebrew University of Jerusalem, Jerusalem 91120, Israel
3 School of Software and Electrical Engineering, Azrieli College of Engineering, Jerusalem 9103501, Israel; noace@jce.ac.il
4 Department of Bioinformatics, Jerusalem College of Technology, Jerusalem 9548370, Israel; mosh9900@gmail.com
5 Unit for Nano Characterization, The Harvey M. Krueger Family Center for Nanoscience and Nanotechnology, The Hebrew University of Jerusalem, Edmond J. Safra Campus, Jerusalem 9190401, Israel; vitalyg@savion.huji.ac.il
* Correspondence: rawi.assad@mail.huji.ac.il
† These authors contributed equally to this work.

Supplementary Data

1. *Early SYTO9/PI and membrane potential*

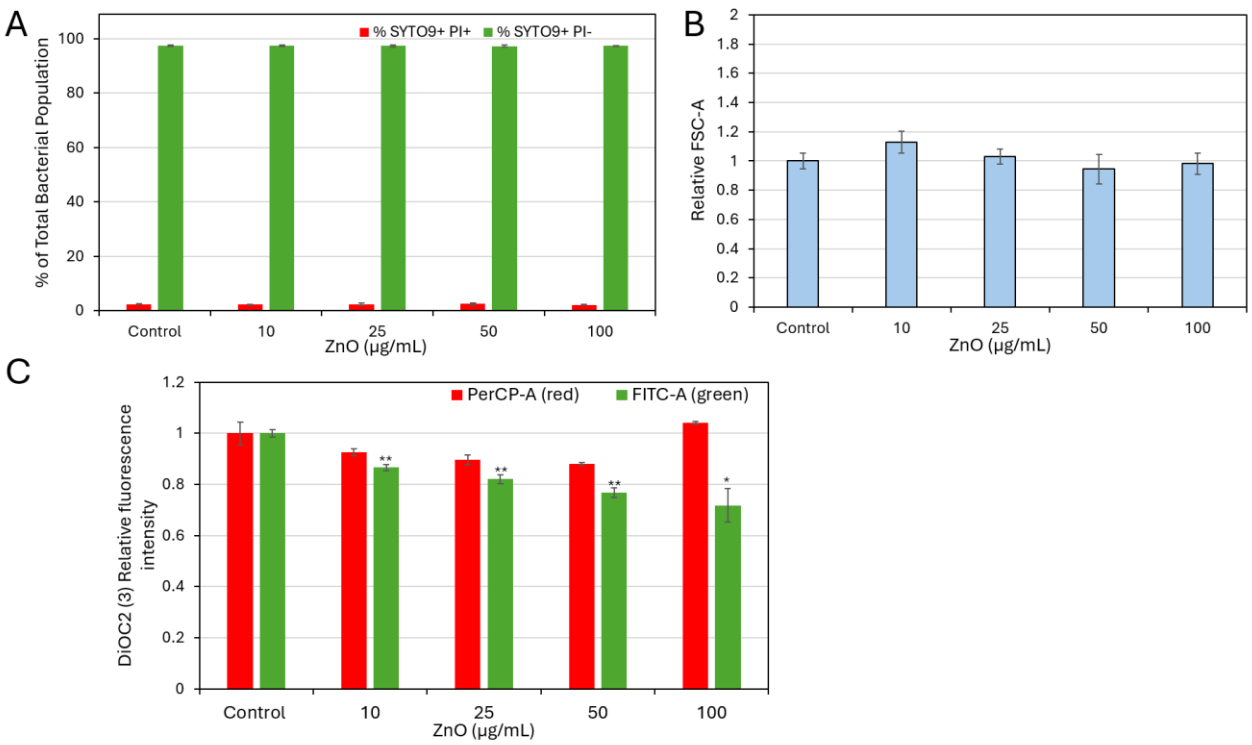

Supplementary Figure S1. ZnO NPs does not alter membrane integrity, and slightly induced membrane potential in *S. sanguinis* after a 2 h exposure to ZnO NPs at 25-500 µg/mL. (**A**) SYTO 9/PI staining of *S. sanguinis*. (**B**) Relative FSC-A values of *S. sanguinis;* and (**C**) Membrane potential assessed using the DIOC2(3) dye. A higher red fluorescence intensity in comparison to green fluorescence intensity indicates membrane hyperpolarization. Data present mean ± SD of triplicate experiments. **p* < 0.0041, ***p* < 0.001 compared to control.

2. *FSC-A after a 24 h incubation with ZnO NPs*

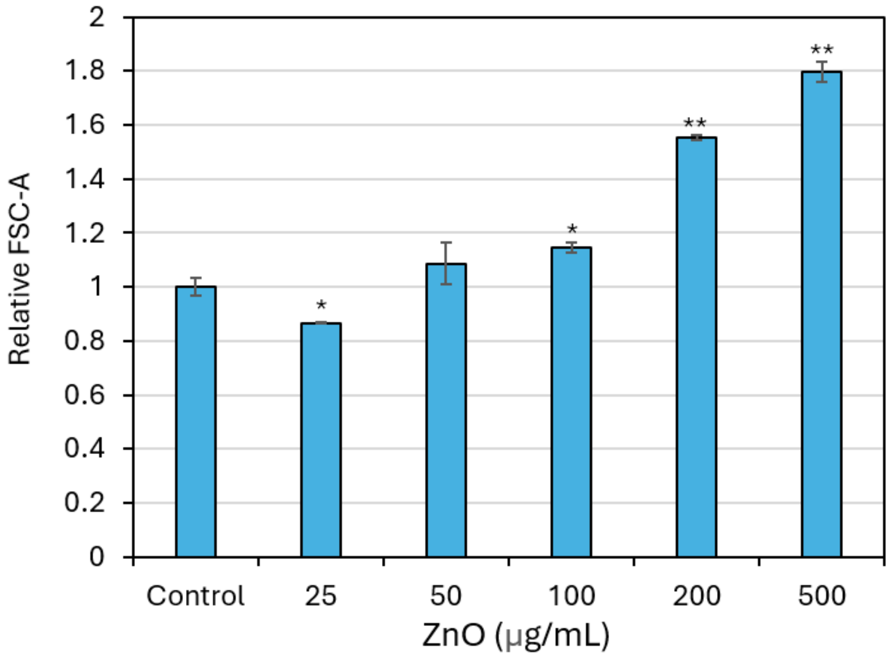

Supplementary Figure S2: Relative FSC-A values of *S. sanguinis* after a 24h incubation to ZnO NPs. An increase in FSC-A is indicative for increased cell size and/or changes in bacterial morphology. Data present mean ± SD of triplicate. **p* < 0.01, ***p* < 0.001 compared to control.

3. *HR-SEM planktonic after 2 h incubation*

HR-SEM imaging was performed to characterize the morphology of S. sanguinis in the planktonic growth phase following ZnO NP exposure. After a 2 h treatment, HR-SEM images at 20,000× magnification revealed that bacteria largely retained their characteristic ovoid morphology and smooth surfaces with intact ovoid morphology and smooth surfaces, similar to untreated controls (Supplementary data S3A-D). A notable exception was observed at 100 μg/mL ZnO NPs (Supplementary data S3E), where a reduction in surface folding was noted, suggesting a potential interference with septum formation at this early stage.

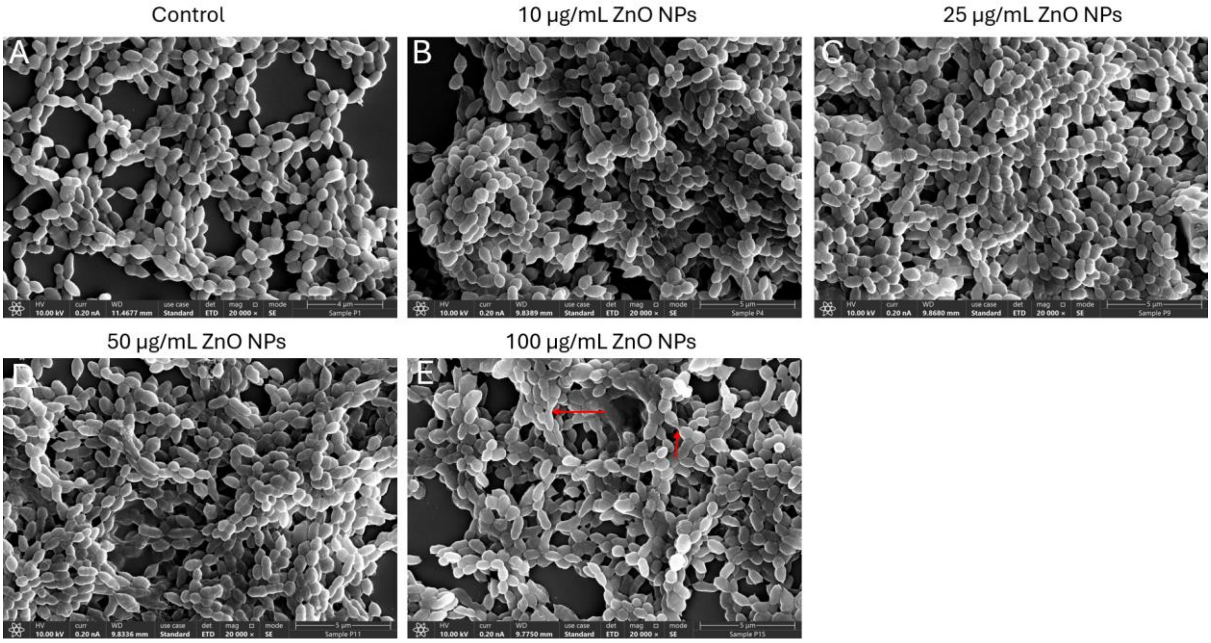

Supplementary Figure S3: High-resolution scanning electron microscopy (HR-SEM) images of planktonic growing S. sanguinis following a 2 h incubation with increasing concentrations of ZnO NPs. (A) control, (B) 10 µg/mL, (C) 25 µg/mL, (D) 50 µg/mL and (E) 100 µg/mL ZnO NPs. Images were acquired double-blinded at 20,000× magnification. Red arrows point to membrane folding representing septa less visible.

4. *Polynomial Surface Fitting for Bacterial Texture Analysis*

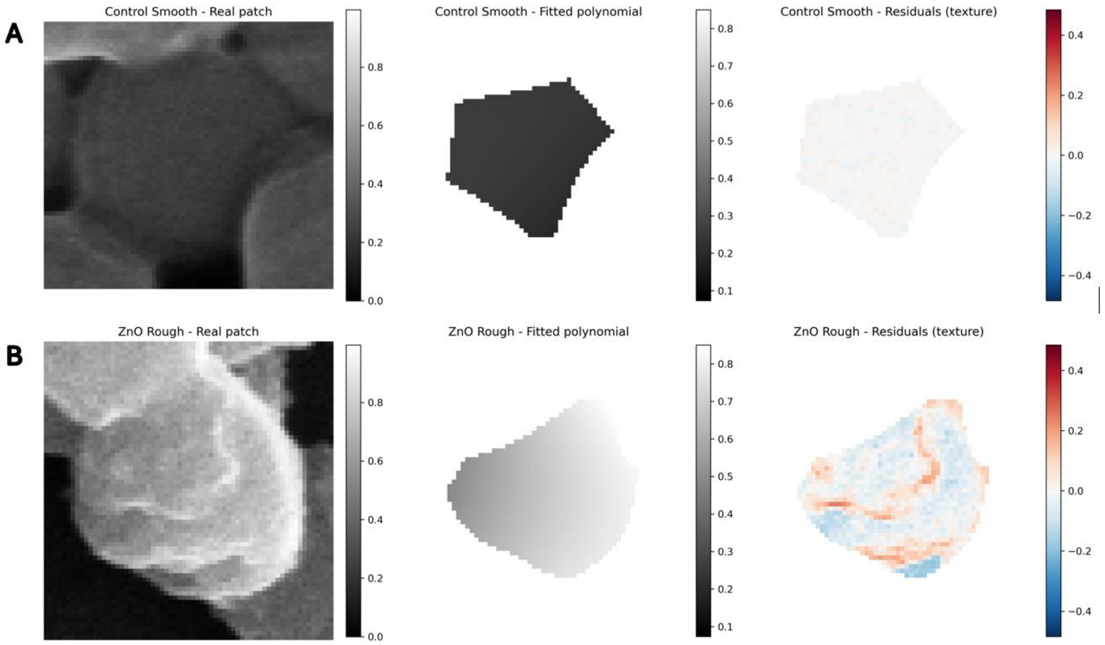

Supplementary Figure S4: Representative examples showing the texture analysis pipeline for (A) a smooth control bacterium and (B) a rough ZnO-treated bacterium (200 µg/mL). For each bacterium: Left panel shows the grayscale intensity patch extracted from the HR-SEM image; Middle panel shows the fitted second-order polynomial surface; Right panel shows the residuals after polynomial subtraction, where red/blue colors indicate positive/negative deviations from the fitted

surface. The control bacterium exhibits minimal residual variation, while the ZnO-treated bacterium shows pronounced residual patterns, reflecting membrane damage and increased surface roughness.

5. Quantitative Morphometric Analysis of Bacterial Surface Texture

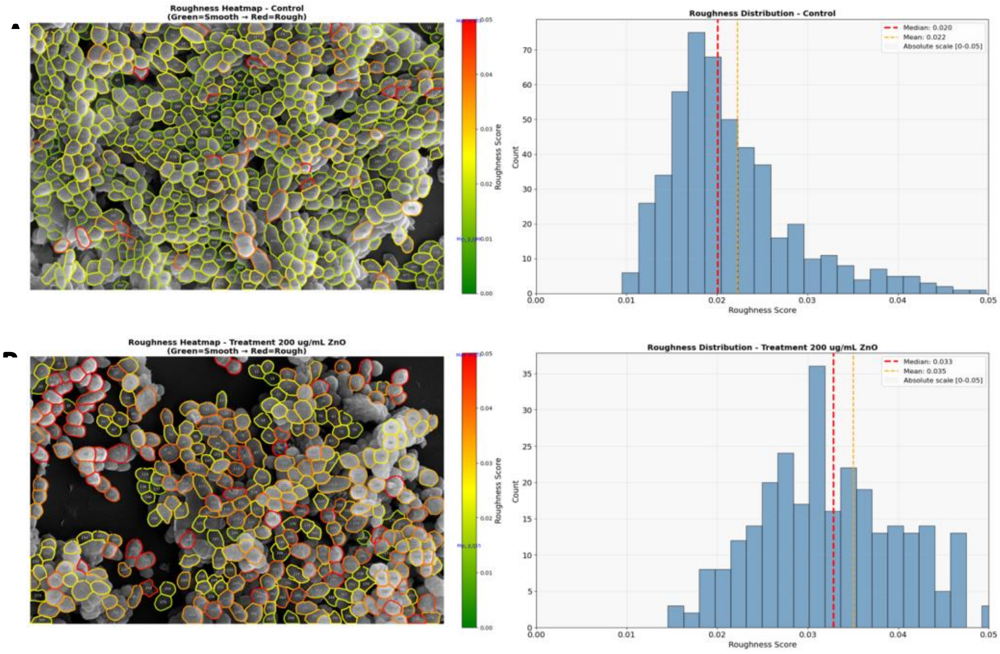

Supplementary Figure S5: Representative HR-SEM images (×20,000 magnification) with automated bacterial segmentation and roughness quantification for (A) Control and (B) 200 µg/mL ZnO NPs treatment. Left panels: Individual bacteria outlined and color-coded by surface roughness score (green = smoother surface; red = rougher, damaged surface) Right panels: Distribution histograms of roughness scores showing median (red dashed line) and mean (orange dashed line). Treatment with 200 µg/mL ZnO NPs shifted the distribution toward higher roughness values, reflecting membrane disruption and surface damage.
